# Attenuating Photostress and Glare Disability in Pseudophakic Patients through the Addition of a Short-Wave Absorbing Filter

**DOI:** 10.1155/2015/607635

**Published:** 2015-03-09

**Authors:** Billy R. Hammond

**Affiliations:** Vision Sciences, Brain and Behavioral Sciences, University of Georgia, Athens, GA 30602, USA

## Abstract

To evaluate the effects of filtering short wavelength light on visual performance under intense light conditions among pseudophakic patients previously implanted with a clear intraocular lens (IOL). This was a patient-masked, randomized crossover study conducted at 6 clinical sites in the United States between September 2013 and January 2014. One hundred fifty-four bilaterally pseudophakic patients were recruited. Photostress recovery time and glare disability thresholds were measured with clip-on blue-light-filtering and placebo (clear; no blue-light filtration) glasses worn over patients' habitual correction. Photostress recovery time was quantified as the time necessary to regain sight of a grating target after intense light exposure. Glare disability threshold was assessed as the intensity of a white-light annulus necessary to obscure a central target. The order of filter used and test eye were randomized across patients. Photostress recovery time and glare disability thresholds were significantly improved (both *P* < 0.0001) when patients used blue-light-filtering glasses compared with clear, nonfiltering glasses. Compared with a nonfiltering placebo, adding a clip-on blue-absorbing filter to the glasses of pseudophakic patients implanted with clear IOLs significantly increased their ability to cope with glare and to recover normal viewing after an intensive photostress. This result implies that IOL designs with blue-light-filtering characteristics may be beneficial under intense light conditions.

## 1. Introduction

The transmission of light energy drastically increases following cataract extraction in the aphakic or pseudophakic eye. Conventional intraocular lenses (IOLs) tend to block only ultraviolet light wavelengths (≤400 nm), but unlike the natural crystalline lens, they do not block light in the short-wave visible region of the spectrum [1] commonly referred to as the blue-light spectrum. Blue-light-filtering (BLF) IOLs were originally designed to reduce this increase in actinic light exposure. A large body of data [2] dating back to Ham's original studies in 1976 [3] suggests that visible blue light is associated with special risks (e.g., photooxidative damage) [4, 5] when the blue-light blocking normally provided by anterior structures of the eye is lost. Without natural blue-light-blocking mechanisms, the blue light that reaches the retina is sufficiently energetic to initiate oxidative damage, and the retina/retina pigment epithelium complex contains high amounts of photosensitizers (e.g., lipofuscin) with an action spectrum that matches the waveband of approximately 400 to 500 nm (hence, the description of the “blue-light hazard”).

Another ramification of filtering light in the visible spectrum is that it alters the incoming stimulus and changes visual function, as has been demonstrated by studies using psychophysical methods to measure the optical density of naturally occurring intraocular BLFs like the anterior lens [6] or macular pigment [7]. Such filtering has a practical advantage in improving vision in photopic conditions in a variety of species, including humans [8, 9]. For example, Wooten and Hammond [10] originally argued that BLF could influence visual range (how far one can see outdoors) by selectively attenuating the deleterious effects of atmospheric blue haze (see the empirical validation of the original modeling by Hammond and colleagues [11]).

Blue-light filtering also reduces the deleterious effects of intense light. Manufacturers of spectacle lenses or sunglasses designed for this purpose face the challenge of reducing glare without reducing visibility. This is often accomplished by using strategies that mimic biological mechanisms for adapting to variations in light intensity. For example, photochromic lenses becoming darker in proportion to light intensity are analogous to how the visual system adjusts sensitivity. Polarizing lenses absorb horizontally oriented glare and pass vertical orientations that tend to not produce glare. Filtering shorter wavelengths tends to also spare visibility since blue light is off the peak of the photopic spectrum. Hence, BLFs reduce retinal exposure to the wavelengths that tend to be the most actinic, photophobic [12], and susceptible to atmospheric (i.e., Rayleigh) scatter while minimally absorbing the medium- to long-wave light that mediates object perception [13].

One assumption inherent in many BLF IOL studies is that the filtering itself is one of the primary factors that produce the visual improvements observed with blue-light-absorbing IOLs. The current study was designed to examine the isolated effects of blue-light filtering. This was done by measuring photostress recovery and glare disability thresholds in pseudophakic patients implanted with clear (i.e., non-BLF) IOLs. A clip-on spectacle lens with filtering characteristics matched to a commonly used BLF IOL (the AcrySof Natural IOL; Alcon Laboratories, Inc.) was then compared to a matched clear lens in a within-patient, same-eye crossover design (see transmission spectra [1, 14]).

## 2. Methods

### 2.1. Study Design and Patients

This was a prospective, randomized, patient-masked crossover study conducted at 6 clinical sites in the United States from September 2014 to January 2014 (This trial is registered with Clinicaltrials.gov NCT01938989). The study consisted of a single visit.

The participating clinicians (listed in the acknowledgments) preselected candidates who fit several criteria. Eligible patients were bilaterally pseudophakic and ≥3 months postimplantation with clear IOLs. Patients were required to be ≥21 years of age, be in good ocular health (based on a clinical interview), and be able to adequately participate in the psychophysical testing. Patients with ocular pathology, degeneration, or media opacity that could have affected study assessments were excluded. Slit-lamp examinations were used to confirm the presence of clear ocular media and the absence of clinically significant posterior capsule opacification. Patients were excluded from participation if they had any conditions that could be exacerbated, triggered, or worsened by exposure to high-intensity light.

Patients were randomized to the order of use of BLF and non-BLF (clear) clip-on glasses, which were worn over patients' habitual correction. Equivalence of the transmission spectrum of the BLF glasses with the natural chromophore used in a commercially available IOL (AcrySof Natural; Alcon Laboratories, USA) was confirmed before use in the study. The BLF and non-BLF clip-on glasses at each study site were reused for all patients evaluated at that site. Patients were masked to the identity of the test and control clip-on glasses. One eye per patient was randomly selected for testing.

The primary efficacy measure was photostress recovery time with the BLF versus non-BLF clip-on glasses. Supportive efficacy measures included glare disability threshold, monocular corrected visual acuity as assessed using the 100% contrast ETDRS chart under photopic lighting, and pupil size. Baseline measures included manifest refraction and uncorrected and corrected distance visual acuity.

The study protocol was approved by an independent ethics committee and Institutional Review Board (Aspire IRB; Santee, CA, USA) for all participating clinical sites. The experimental procedures adhered to the tenets of the Declaration of Helsinki. Patients were informed about the aims and methods of the study, and all patients signed a statement of informed consent.

### 2.2. Equipment and Procedures

**Figure 1:**
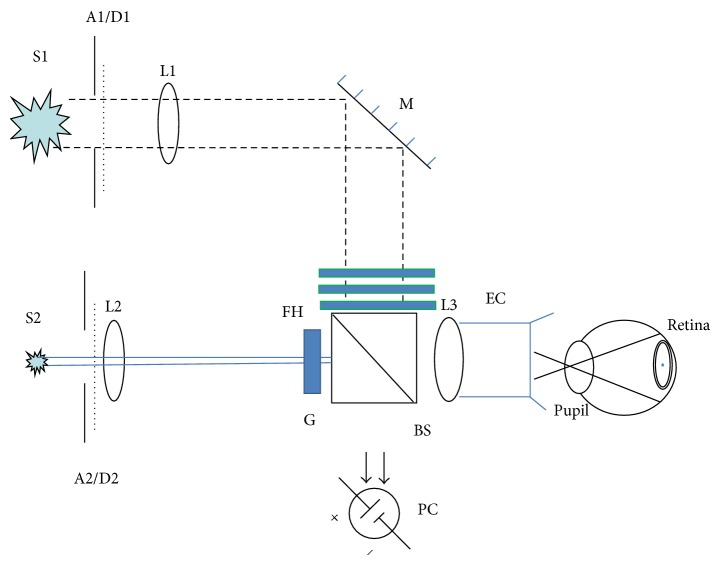
Schematic of the optical system used to measure disability glare thresholds and photostress recovery time. A1-A2: apertures; BS: beam splitters; D1-D2: diffusers; EC: final focusing lens and eye cup for head positioning; FH: filter holder; G: grating and aperture for defining target; L1–L3: planoconvex achromatic lenses; M: mirror; PC: photocell; S1-S2: LED light sources.

The apparatus used to measure glare disability and photostress recovery time was a 2-channel Maxwellian view system and is shown in Figure 1. The apparatus used white LEDs (LXML-PWN2, 4100 K; Phillips LumiLeds Lighting Company, San Jose, CA, USA) that provided a relatively broad spectrum of energy (major peaks at 445 and 570 nm) as the glare source.

The intensity of this glare source was electronically controlled (via pulse-width modulation) with customized computer software. One channel presented a target which was a 1°-diameter disk (peak wavelength, 570 nm) containing a contrast grating stimulus (4 cycles per degree). The luminance of the bars within the grating was 0.1 candela/m^2^. This target stimulus was used for both the glare disability and photostress measurements. To aid patients in maintaining the same position during the procedure, an eyepiece with a soft rubber eye cup was used. Prior to testing, patients were aligned to the optical system (this was facilitated by the structure of the eye cup). The BLF- and non-BLF-absorbing filters that we tested were incorporated into the eyepiece so that they were not visible to the patient. Careful adjustments were made such that the image was in focus and in the plane of the patient's pupil.

When testing photostress recovery, the 1° target stimulus was shuttered at 500 ms on and off and a second channel provided a photobleaching light of high intensity (corneal irradiance, 5 log Trolands). The photobleaching light was presented for 5 seconds. The patients were informed before the photostressor appeared and were instructed to keep their eyes open for the duration of the exposure. At the end of the 5-second exposure, timing began to determine the length of time required until the target stimulus became visible again. At the point of reemergence, the participant pressed a button and the timing stopped. There were 3 repetitions separated by a waiting period of ≥2 minutes for each patient.

When testing glare disability, the target stimulus was presented at 2 seconds on and 1 second off. A second channel provided an annulus with an 11-degree inner diameter and 12-degree outer diameter. Before each trial, the annulus was set at a level well below that which would cause the target stimulus to be veiled. The intensity of the annulus was then adjusted by the experimenter until the patient indicated verbally that the target stimulus was no longer visible. This procedure was repeated for a total of 5 measurements (recorded as time in seconds), and patients were instructed to maintain their criterion threshold across trials. Five measurements were planned to be recorded per eye, unless a patient's values had more than ~5% variability among measurements, in which case up to 4 additional measurements were conducted.

Careful calibrations were conducted in this study to ensure that the stimuli did not vary across sites. Hence, both radiometric and photometric calibrations were regularly performed. Prior to each experimental sitting, a dedicated radiometer was used to ensure that total light output remained constant (S370 Optometer with a PIN-10 photohead, UDT Instruments, Hawthorne, CA, USA). Photometric calibrations were done using a telescopic spectral radiometer (model PR650, PhotoResearch Inc. Chatsworth, CA, USA) with the stimuli projected onto a white reflectance standard calibrated to the instrument. Spatial alignment of the channels was checked every session by increasing the intensity of the light source and checking the precise location of the projected image against a fixed plate on the optical table.

### 2.3. Statistical Analysis

Endpoints were analyzed in the efficacy analysis data set, which consisted of patients who provided data on ≥1 of the efficacy endpoints.

The arithmetic mean photostress recovery time with BLF versus non-BLF glasses was compared using a 1-sided paired *t*-test. The difference between arithmetic means, 1-sided 95% CI, and *P* values were calculated. The arithmetic mean of glare disability threshold (*X*) for each patient was transformed by solving for *Y* in the equation *Y* = 4.89 + 5.43*X*. The resulting values were log_10_ transformed. Glare disability threshold data were compared for BLF versus non-BLF glasses using paired *t*-tests. Corrected visual acuity, pupil size, and demographic/baseline characteristic data were summarized descriptively.

Assuming a log-transformed photostress recovery time SD of 0.35, a minimum sample size of 153 patients was determined to provide 80% power to detect a 20% difference in photostress recovery time.

## 3. Results

One hundred fifty-four of 156 enrolled patients completed the study (97.5%). One patient was invalidated because they violated inclusion/exclusion criteria (implantation with a BLF IOL). Nine other patients had incomplete data sets due to physical limitations or inability to maintain alignment with the optical system. Most patients were white (94.9%) and there were more women (58.3%) than men (41.7%). Details regarding the patients tested in this study are listed in Table 1.

Mean photostress recovery time was significantly lower when patients were wearing BLF compared to non-BLF clip-on glasses (*P* = 0.0001; 95% CI, −2.08 to −0.66 seconds; Table 2). The mean ± SD difference between BLF and non-BLF glasses was −1.4 ± 4.3 seconds. Glare disability thresholds were significantly higher (i.e., patients could tolerate more light before losing sight of the central grating target) with BLF versus non-BLF glasses (*P* = 0.00014; 95% CI, 0.06–0.18). The difference between BLF and non-BLF glasses was 0.12 ± 0.38 log units.

Corrected visual acuity was comparable with versus without BLF. Mean ± SD acuity was 0.051 ± 0.105 logMAR with the BLF clip-on glasses (range: −0.30 to 0.34 logMAR) and 0.049 ± 0.099 logMAR with the non-BLF clip-on glasses (range: −0.3 to 0.38 logMAR). Mean ± SD pupil size was 3.54 ± 0.80 mm and 3.52 ± 0.79 mm with the BLF and non-BLF clip-on glasses, respectively.

## 4. Discussion

This study was aimed at assessing the role of additional blue filtration on vision when assessed under intense light conditions. Patients with pseudophakia with clear implants had a short-wave filter placed in front of their eyes which had absorption characteristics matching the young natural crystalline lens [1, 14]. This filtering lens was placed behind optical baffling and an eye cup so that patients would remain blinded to the comparison with a lens transparent to visible light. The order of testing conditions was randomized. Consistent with past studies (see Table 3) that have compared clear and BLF IOLs, we found a visual advantage of BLFs when testing glare disability and photostress recovery. Blue-light filtration increased tolerance to veiling white light and significantly lowered glare disability thresholds. For example, the amount of energy (i.e., light intensity) required to veil the central target in the glare disability assessment was about 23% higher when using the BLF lens compared to the clear lens (this percentage is based on a linear translation of the logged values). Photostress recovery time was improved by about 28%. These changes could translate to meaningful differences in everyday life. As a practical example, if someone is driving 60 mph and is blinded by the sun or bright headlights, a 10-second photostress recovery time translates to about 880 feet traveled before normal visual function is recovered. Regaining visual function 28% more quickly means seeing about 246 feet sooner (about two thirds the length of a football field).

The mechanism for how blue-light filtering reduces photostress is straightforward: it simply reduces the intensity of the exposure and, hence, decreases recovery time. Photostress is caused by short intense light exposure (which is particularly damaging when dark adapted due to higher levels of photosensitizing photopigment [4]). Such exposure causes adaptive change and photopigment isomerization which results in temporary loss of vision. Endogenous BLF mechanisms such as the natural crystalline lens or macular pigment and artificial mechanisms such as BLF IOLs can absorb the incoming light and the forward scatter reducing the resultant loss of vision. This filtering protection, which operates over short exposures to intense light, does not lower overall sensitivity since the visual system adjusts sensitivity to offset stable changes in illumination [39, 40].

The mechanism for glare disability is less straightforward. It has been argued [41, 42], for instance, that intraocular filters (macular pigment or BLF IOLs) do not reduce glare disability. The basis for this argument is ecological; to wit, the authors note that the empirical studies that have found that BLF IOLs or macular pigment reduce glare disability have done so because they are “…biased by using bluer glare than target illumination, guaranteeing that glare light is preferentially reduced by yellow chromophores. Headlights and sunlight cause glare and illuminate targets. That's why clinical glare tests use glare and target illumination with similar spectra simulating real-world conditions” [42].

The question is whether similar spectra really simulate real-world conditions; we would argue that they do not. Unless a target (an object in the patient's line of sight) reflects all wavelengths equally (unlikely unless it is a mirrored surface or a perfect white), it would never have the same spectrum as a glare source such as the sun. Perhaps a more reasonable framing would be whether it is likely that a glare source (like the sun) would have more short-wave energy when compared to a probable target (hence, favoring a yellow intraocular filter). This is likely for several reasons.

1. The most common source of glare is the white light of the sun, which has a strong short-wave component [43].
2. Glare sources often come in from the side or above, whereas targets are, by definition, objects that are within our line of sight. Hence, even under the highly unlikely circumstance that the glare source and target share the exact same spectra, the composition would not be the same at the plane of the retina. This is because of the differences in light path [10]. Light reflected from an object within our line of sight passes through the atmosphere and short-wave energy scatters out of the light path (Rayleigh scatter does the opposite for objects in the surround).
3. Target stimuli are often composed of medium-wave light. One argument for the evolution of spectral sensitivity that peaks in the medium-wave region of the visible spectrum is that such a spectrum matches objects that are commonly perceived in the environment [44]. Many of the major pigments throughout nature are represented in the medium-and-longer-wave region. For example, chlorophyll is green and most of the carotenoids are yellow, orange, and red (few pigments one sees in a natural landscape are blue).
4. The light stimulus for S-cones (i.e., blue-light-sensing cones), is largely filtered by yellow intraocular filters, which are relatively sparse, contribute little to the luminosity function, and mostly mediate color perception [45]. Spatial vision is largely mediated by medium- and long-wave cones (this spectra tuned to match ecological condition).

Based on these reasons, it could be argued that many of the available clinical glare tests that match the spectra of the glare source and target are not ecologically valid (rather, they are designed to diagnose clinical conditions). The best way to measure glare disability is to use a glare source that matches a source that is commonly encountered, such as the sun, and to use a target that is strongly at the peak of the photopic spectral sensitivity curve, thereby matching the spectral content of most objects one would view.

Several empirical studies have examined the effects of macular pigment and BLF IOLs on visual function under glare conditions (see Table 3).

Most of the BLF IOL studies have used a case-control design, with the exception of Hammond et al. (2010) [18], which compared glare disability and photostress recovery using a contralateral design where visual function was tested in 1 eye implanted with a BLF IOL compared to the other eye with a clear IOL. That study found significant visual benefit in the eye with the blue-light-filtering IOL. Hammond and colleagues [17, 18] and Gray and colleagues [15, 16] used stimuli or test circumstances that closely match real-world scenarios; in the study by Hammond, visual stimuli were matched to daytime sunlight, and Gray tested patients using a driving simulator. In these studies, there was a clear benefit of the BLF IOLs with regard to photostress recovery and glare disability thresholds, similar to the findings of the current study. The results of other studies are largely mixed. Niwa et al. [22] and Pandita et al. [23] reported significant improvements using a BLF IOL when contrast sensitivity was measured under glare conditions, whereas K. Hayashi and H. Hayashi [19], Muftuoglu et al. [20], and Neumaier-Ammerer et al. [21] found no difference in glare disability threshold or contrast sensitivity function between clear and BLF IOLs. However, these studies used light sources without a significant blue-light component and/or glare devices without strong discriminative ability [38].

The use of clip-on glasses worn over pseudophakic patients' habitual correction is a potential limitation of this study. Additionally, a single light source was used and psychophysical testing was not performed under varied light conditions (e.g., mesopic and scotopic).

In this study, photostress recovery time and glare disability thresholds were significantly reduced by blue-light filtration, whereas visual acuity was not compromised. Our findings are consistent with the visual benefit one might predict from simply returning the eye closer to its natural state, in this case, the young natural crystalline lens. Yellow (i.e., blue-light filtering) chromophores added to an IOL may help ameliorate complications of photostress and glare disability.

## Figures and Tables

**Table 1:** Patient demographics and baseline characteristics.

Parameter	Patients (*N* = 156)
Age, y	
Mean ± SD	69.8 ± 8.0
Range	48–88
Sex, *n* (%)	
Female	91 (58)
Male	65 (42)
Race, *n* (%)	
White	148 (95)
African American	5 (3)
Asian	1 (1)
Pacific Islander	1 (1)
Other	1 (1)
Pseudophakia status, months	
Mean ± SD	16.3 ± 27.6
Range	3–216
Spherical correction, D	
Mean ± SD	−0.121 ± 0.739
Range	−2.75 to +2.25
Cylindrical correction, D	
Mean ± SD	−0.015 ± 0.672
Range	−2.50 to +2.25
Axis,^*^ degrees	
Mean ± SD	55.8 ± 65.4
Range	0–180
Intraocular lenses,^†^ *n* (%)	
Abbott Medical Optics^‡^ (Tecnis)	114 (73)
Lenstec (Lenstec, Softec, and Softec HDO)	22 (14)
Bausch & Lomb (Crystalens and Akreos)	13 (8)
Alcon (SA60AT and SA6003)	3 (2)
Hoya	1 (1)
Missing	1 (1)
Test eye, *n* (%)	
OD	76 (49)
OS	79 (51)
Missing	1 (1)

OD: oculus dexter (right eye); OS: oculus sinister (left eye).

^*^ *n* = 115.

^†^1 patient had 2 different Bausch & Lomb intraocular lenses.

^‡^1 patient had an Abbott Medical Optics multifocal intraocular lens.

**Table 2:** Photostress recovery time and glare disability threshold (efficacy analysis data set).

	BLF Glasses	Non-BLF Glasses
Photostress recovery time, seconds		
*N*	145	144
Mean ± SD	5.66 ± 6.20	6.94 ± 7.16
Range	1.0–29.3	1.0–36.0
Difference (*P* value^*^; 95% CI)	−1.37 (0.00010; −2.08 to −0.66)	
Glare disability threshold, log units		
*N*	146	147
Mean ± SD	1.37 ± 0.88	1.26 ± 0.92
Range	−0.6 to 3.1	−0.6 to 4.0
Difference (*P* value^*^; 95% CI)	0.12 (0.00014; 0.06–0.18)	

BLF: blue-light filtering.

^*^1-sided *P* value from paired *t*-test.

**Table 3:** Studies on blue-light intraocular filters and visual benefit.

Study	Sample	Design	Supplement	Variables	Effect size *P* value (relation to MP, IOL, or sup)
**Blue-light filtering IOLs**					
Gray et al., 2011 [15]	34 adults	Case-control	n/a	GD in a driving simulator	0.0008 versus clear IOLs
Gray et al., 2012 [16]	33 adults	Case-control	n/a	GD in a driving simulator	0.05 versus clear IOLs
Hammond et al., 2009 [17]	58 adults	Case-control	n/a	Glare disability	0.02 versus clear IOLs
Hammond et al., 2009 [17]	58 adults	Case-control	n/a	PS recovery	0.01 versus clear IOLs
Hammond et al., 2010 [18]	52 adults	Contralateral Comparison	n/a	GD	0.04 versus clear IOLs
Hammond et al., 2010 [18]	52 adults	Contralateral Comparison	n/a	PS recovery	0.02 versus clear IOLs
Hammond et al., 2010 [18]	52 adults	Contralateral Comparison	n/a	Chromatic contrast	0.00003 versus clear IOLs
K. Hayashi and H. Hayashi, 2006 [19]	74 adults	Case-control	n/a	CSF under glare	Null^*^
Muftuoglu et al., 2007 [20]	38 adults	Case-control	n/a	GD	Null^*^
Neumaier-Ammerer et al., 2010 [21]	76 adults	Case-control	n/a	CSF under glare	Null^*^
Niwa et al., 1996 [22]	64 adults	Case-control	n/a	CSF under glare	0.025
Pandita et al., 2007 [23]	120 adults	Case-control	n/a	CSF under glare	Photopic = 0.005, Mesopic = 0.01

**Macular pigment**					
Hammond et al., 2013 [24]	150 adults	Cross-sectional	n/a	Glare disability	0.0015
Hammond et al., 2013 [24]	150 adults	Cross-sectional	n/a	PS recovery	0.01
Hammond et al., 2013 [24]	150 adults	Cross-sectional	n/a	Chromatic contrast	0.00005
Hammond et al., 2014 [25]	109 adults	RCT	12 mg/1 year	PS recovery	0.01
Hammond et al., 2014 [25]	109 adults	RCT	12 mg/1 year	GD	0.21
Hammond et al., 2014 [25]	109 adults	RCT	12 mg/1 year	Chromatic contrast	0.03
Kvansakul et al., 2006 [26]	34 adults	RCT	3 arms of L and Z	Intraocular scatter	+ for L (no *P* reported)
Loughman et al., 2012 [27]	36 adults	RCT	24 subjects on L, Z, MZ	VA and CSF measured under glare	0.006
Loughman et al., 2010 [28]	142 adults	Cross-sectional	n/a	VA and CSF measured under glare, PS recovery	Null^*^
Nolan et al., 2011 [29]	121 adults	RCT	13 mg, one year	CSF under glare	0.05
Olmedilla et al., 2003 [30]	17 elderly cataracts patients	RCT	12 mg, 2 yrs	Glare sensitivity	0.005
Renzi and Hammond, 2010 [31]	50 adults	Cross-sectional	n/a	Chromatic contrast	0.0001
Richer et al., 2004 [32]	90 v, dry AMD patients	RCT	3 arms, ~10 mg L, one year	Glare questions	0.10 (ns)
Richer et al., 2011 [33]	60 dry AMD patients	RCT	L 9 mg, one year	Glare recovery	0.01
Stringham and Hammond, 2007 [34]	36 adults	Cross-sectional	n/a	GD, PS recovery	0.0001
Stringham and Hammond, 2008 [35]	40 adults	Intervention (no placebo)	12 mg/6 mos	GD, PS recovery	0.0001
Stringham et al., 2011 [36]	26 adults	Cross-sectional	n/a	CSF measured under glare, PS recovery	0.0001
Yao et al., 2013 [37]	120 adults	RCT	20 mg L, one year	CSF under glare, glare Qs	CSF (0.05), Qs (0.01, 0.03)

CSF: contrast sensitivity function; GD: glare disability threshold; PS: photostress; RCT: placebo-controlled randomized trial.

^*^All patients were adults. These studies used glare sources (halogen or tungsten) with little or no short-wave energy and/or clinical tests with low discriminative ability [38].
